# A unique case of extra-cerebral diffuse glioneuronal tumor with oligodendroglioma-like features and nuclear clusters

**DOI:** 10.1016/j.radcr.2025.04.067

**Published:** 2025-05-12

**Authors:** Wu Maurice, Naderian Ashkun, Iyengar Krishnan, Coulthard Alan, Jeffree Rosalind

**Affiliations:** a Ipswich Hospital Department of Emergency Medicine, Ipswich, Queensland, Australia; b The University of Queensland, Brisbane, Queensland, Australia; c Royal Brisbane and Women's Hospital Department of Medical Imaging, Brisbane, Queensland, Australia; d Royal Brisbane and Women's Hospital Department of Anatomical Pathology, Brisbane, Queensland, Australia; e Alfred Hospital Department of Neurosurgery, Melbourne, Victoria, Australia; f Monash University, Melbourne, Victoria, Australia

**Keywords:** Diffuse glioneuronal tumor, Oligodendroglioma-like features, MRI, DGONC

## Abstract

Diffuse glioneuronal tumor with oligodendroglioma-like features and nuclear clusters (DGONC) is a newly recognized provisional entity in the fifth edition of the WHO Classification of Central Nervous System Tumors, typically found in pediatric patients with supratentorial disease. We describe a male in his forties who presented with recurrent seizures and was found to have a heavily calcified lesion in the left temporal lobe and an additional lesion in the sacral region of the thecal sac; tissue biopsy of the sacral lesion demonstrated an oligodendroglioma-like morphology, strong synaptophysin and OLIG2 expression, and absent widespread GFAP staining, consistent with DGONC. This represents an unusual occurrence of DGONC outside the supratentorial region, with imaging findings that mimicked a chronic inflammatory process. Radiologists should be aware of the potential for DGONC to present in atypical locations and should consider it in the differential diagnosis of apparently non-neoplastic lesions.

## Introduction

Diffuse glioneuronal tumor with oligodendroglioma-like features and nuclear clusters (DGONC) is a new addition as a provisional entity in the WHO Classification of Central Nervous System Tumors fifth Edition in 2021. The majority of DGONC cases occur in pediatric patients, with a median age of 9 years and equal sex distribution, with all described cases thus far being supratentorial in location [1].

We describe the case of a male in his forties who was diagnosed based on morphological features, with a rare case of DGONC outside of the supratentorial region. The lesion was identified on PET and MRI imaging in the sacral region of the thecal sac, initially deemed to exhibit indolent findings.

## Case report

A male in his forties with no previous medical history sustained a seizure while working at his desk job. He was found by co-workers in a postictal state—confused, disoriented, and experiencing sore back muscles—and was brought by an ambulance to the emergency department.

The initial assessment in the emergency department was that of a first seizure episode, with spontaneous resolution with no residual neurological features, and the patient was subsequently discharged and referred to a neurologist, with a prescription for levetiracetam, although medication hesitancy resulted in the patient not filling the script. He suffered a second tonic-clonic seizure approximately 3 months later, which self-resolved with no residual neurological deficits. Following this episode, the patient was compliant with the levetiracetam prescription.

As part of the seizure work-up, blood cultures and lumbar puncture for CSF samples were performed, which returned negative cultures and negative cytology.

**Fig. 1:**
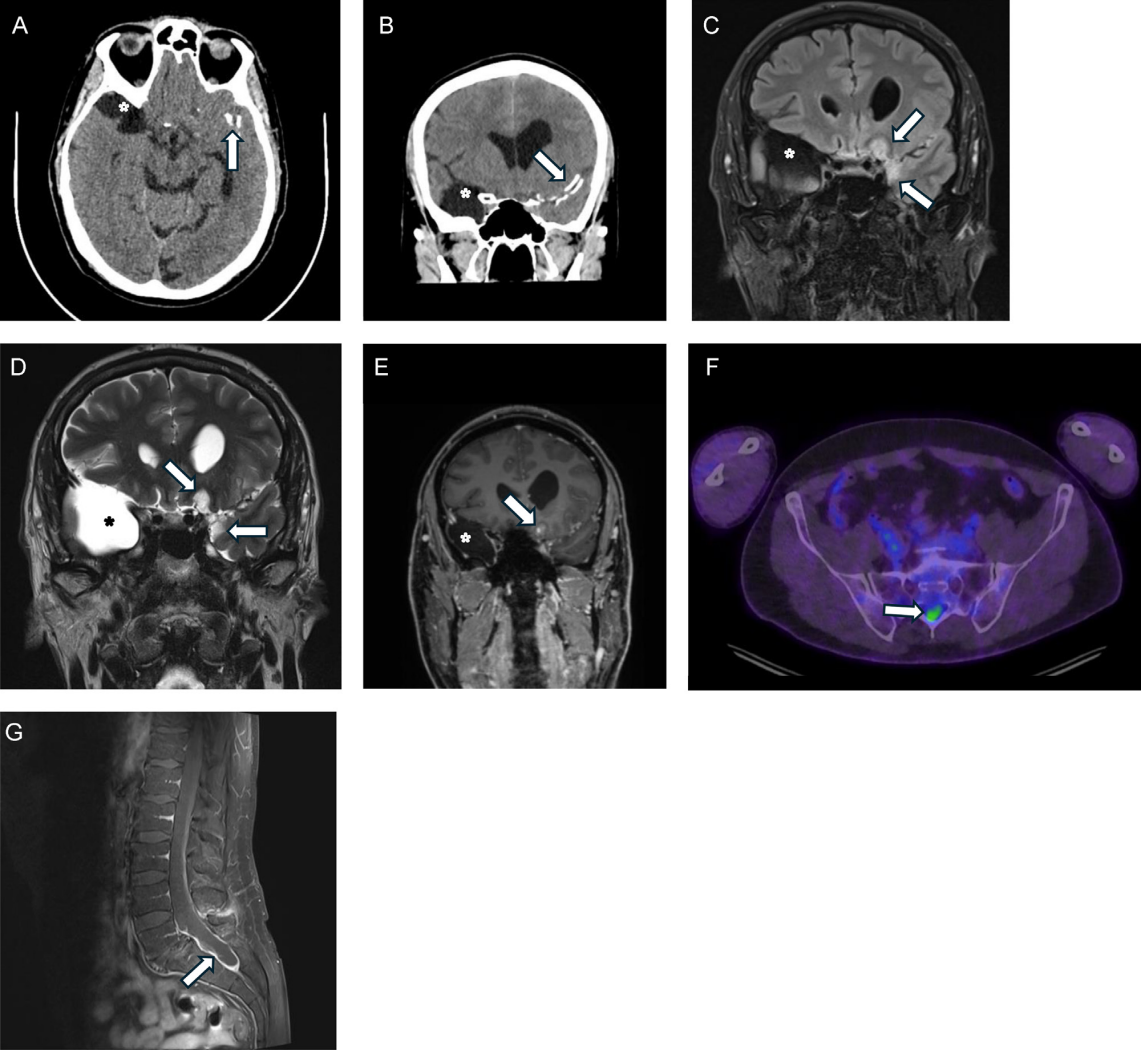
CT head axial (A) and coronal plane (B) sections demonstrating calcification along the left sylvian fissure (arrows). Incidental right temporal pole arachnoid cyst (asterisks) on MRI and CT (A-E). Coronal FLAIR (c) and T2 weighted MRI (D) show heterogenous hyperintensity involving medial left temporal and frontal lobes (arrows), with widening and displacement of the left olfactory sulcus. There is patchy leptomeningeal enhancement (arrow) after intravenous contrast on coronal T1-weighted section (E). An axial plane section from the FDG-PET study at the level of the pelvis (F) demonstrates abnormal tracer uptake within the sacral spinal canal (arrow). Sagittal plane fat-suppressed T1 MRI acquired after IV contrast (G) demonstrates distension, diffuse thickening and hyperintensity of the sacral thecal sac (arrow).

A series of imaging was also performed: CT head, whole-body FDG-PET scan, brain MRI, and lumbosacral MRI. On the CT head (Fig. 1A, B), calcific densities over the left Sylvian fissure in the left temporal lobe were identified, with an appearance favoring a long-standing or congenital abnormality or a healed infection from childhood. MRI of the head with gadolinium contrast identified an enhancing extra-axial mass over the left temporal region with heavy calcification (Fig. 1C-E). A PET scan was also ordered; notably, a new area of abnormal FDG uptake was identified in the sacral spinal canal (Fig. 1F). As such, MRI was performed on the spine, identifying a lesion located in the caudal aspect of the thecal sac in the lumbosacral region (Fig. 1G). The appearance of this lesion was deemed to be akin to a chronic inflammatory process.

After consultation with the neurosurgical unit and shared decision-making, the patient was counselled on possible options to obtain a tissue diagnosis. Biopsy of the sacral dura was opted for over temporal lesions owing to a better safety profile. The patient recovered without any complications.

**Fig. 2:**
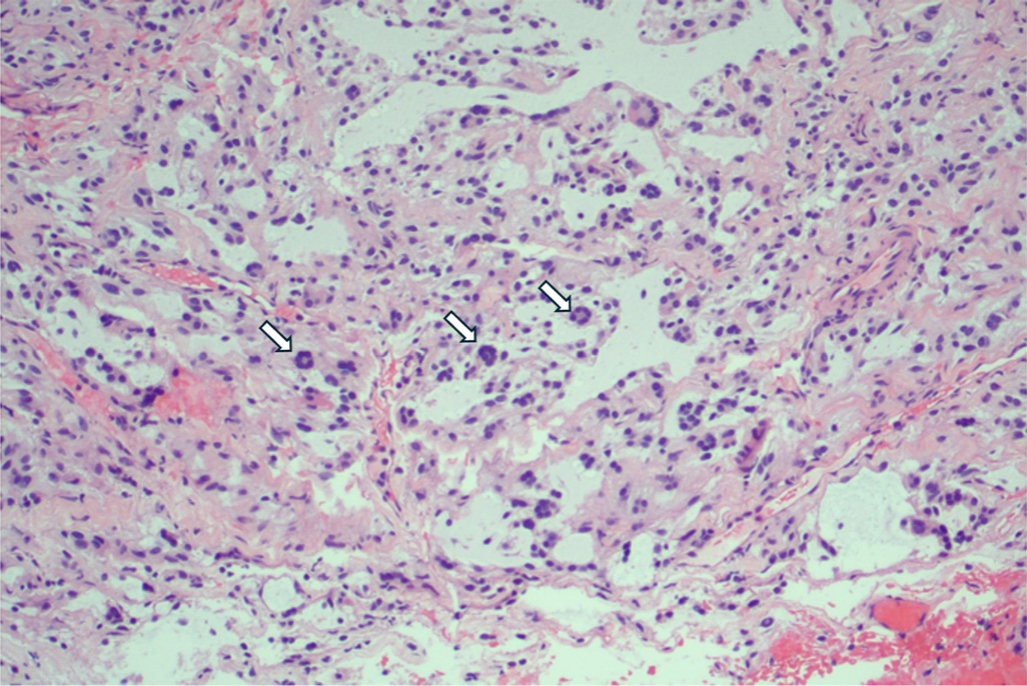
H&E stain at 100x magnification of the sacral lesion, morphologically consistent with DGONC, demonstrating characteristic nuclear clusters (arrows).

The biopsied tissue (Fig. 2) demonstrated an oligodendroglioma-like appearance under microscopy, with no 1p/19q co-deletion or BRAF rearrangement. The positive markers included synaptophysin(strong), S100 (strong), OLIG2 (strong), GFAP (focal), map2 (weak), and ATRX (normal reaction). The following markers were negative: chromogranin, EMA, cytokeratin (AE1/AE3), IDH1-R132H, and BRAF-V600E. P53 expression was wild-type, and Ki67 expression was low (<1%). The characteristics of the tumor were reasonably novel and required consultation with a second neuropathologist, and the tumor initially conferred diffuse glioneuronal leptomeningeal tumor as the main differential diagnosis.

The patient was then referred to a medical oncologist who commenced treatment with temozolomide. The patient has since been followed-up by his medical oncology physicians for 3 years and has remained seizure-free with no new symptoms and a stable disease appearance radiologically.

Following the publication of the WHO Classification of Central Nervous System Tumors fifth Edition in 2021, this case was revisited. With findings of cells exhibiting oligodendroglioma-like appearance, strong OLIG2 and synaptophysin expression, and absence of widespread GFAP expression, the diagnosis was revised to DGONC.

## Discussion

This is an unusual case of a DGONC found outside the supratentorial region.

In order to compare our tumor to existing cases, we conducted a title and abstract search looking for case reports and case series across 5 databases (PubMed, Medline, Embase, Web of Science, Cochrane) in April 2025 using the following strategy: (dgonc[tiab]) AND (((“diffuse glioneuronal”[tiab]) AND (oligodendroglioma[tiab])) AND (“nuclear clusters”[tiab])). From this, we identified 45 articles, with 15 remaining after de-duplication, of which 8 relevant articles remained, comprising case series, case reports, and analysis of tumor database, encompassing a total of 48 unique cases [[2], [3], [4], [5], [6], [7], [8], [9]]. We then cross referenced this with the 5^th^ edition of the WHO Classification of Central Nervous System Tumors, which utilizes results from Deng et al [2] and Pickles et al [4], of which there is a median age of 9 years with a wide age range with 1 patient aged 75 years, from 23 patients where age of disease onset was known, with equal sex distribution. The remainder of articles encompassed 14 cases [3,[5], [6], [7], [8], [9]], with a mean age of 11.2, median age of 8.75, with an age range of 4.2 years to 32 years, of which only 2 cases were adults (age greater than 18 years) which highlights a propensity for this tumor to affect the pediatric population. There were no cases with documented occurrence of the tumor outside of the supratentorial region.

Based on our review of the literature, our case of DGONC would be unique in being the only known case with disease occurrence outside of the supratentorial region. In addition, it is also an unusual case due to it being identified in the patient’s forties, compared to most cases which occur in a pediatric population.

It should be noted that our case of DGONC was diagnosed based on the morphological features. Based on the current classification criteria, the essential features of DGONC include the methylation profile of DGONC, nuclear clusters exhibiting oligodendroglioma-like morphology, strong expression of OLIG2 and synaptophysin, and absence of widespread GFAP expression, with the caveat that morphological features can be used for approximation in the absence of methylation; monosomy 14 is listed as a desirable but nonessential feature [1]. As our case demonstrated cells exhibiting oligodendroglioma-like appearance, strong OLIG2 and synaptophysin expression, and absence of widespread GFAP expression, the diagnosis of DGONC was assigned based on these morphological features. By the time we revisited the case, methylation profiling and karyotyping could no longer be performed on the remaining salvaged material.

Radiological findings in this case of DGONC are unusual for neoplastic processes but are consistent with the imaging findings of previously reported cases of DGONC. The temporal lesion, which is deemed to be a second focus, demonstrates heterogeneous hyperintensity on T2 weighted imaging, with patchy enhancement with gadolinium contrast, and no restricted diffusion, which appears to be typical in DGONC, but we acknowledge a paucity of cases described in the literature [9]. Heavy calcification was present in the lesion (Fig. 1A, B), which favored a granulomatous or infectious process. We also note that restricted diffusion has been observed in 1 case of progressive disease; however, our case demonstrated a stable disease process over the course of 3 years.

With respect to the sacral focus, the findings are completely novel in the case of DGONC, as no extra-cranial disease has been reported in the literature. The sacral focus on MRI had findings unusual for a neoplastic process, with nonspecific thickening of the thecal sac with enhancement, more consistent with a chronic inflammatory process as opposed to neoplasm.

Currently, there is no consensus on the ideal treatment. Our patient had only undergone biopsy of the sacral lesion, was subsequently treated with temozolomide, and experienced a stable disease course for both cerebral and spinal lesions. Other patients have undergone a combination of resection with chemotherapy and radiotherapy, with successful remission or stabilization of the disease course [10]. One patient had stable disease after resection with no chemotherapy or radiotherapy treatment [5].

## Conclusion

At time of writing, DGONC remains a provisional entity in the WHO Classification of Tumors. We have described an unusual case of DGONC outside the supratentorial region, diagnosed based on morphological features, with both supratentorial and sacral foci.

We also highlight that with regards to DGONC, cerebral lesions on CT and the sacral lesions on MRI can mimic non-neoplastic processes such as chronic inflammation. As such, clinicians should be aware of such radiological appearances and remain vigilant that neoplastic processes remain a differential.

## Patient consent

Informed written consent was obtained from the patient and they have given approval for the publication of this case report and accompanying images.
